# Isolation, identification of porcine a rotavirus, and preparation of the monoclonal antibodies

**DOI:** 10.3389/fvets.2025.1689520

**Published:** 2025-10-20

**Authors:** Yu Hu, Hongjin Wu, Boqian Zha, Deping Song, Xiangdong Wu, Huansheng Wu

**Affiliations:** 1 Department of Veterinary Preventive Medicine, College of Animal Science and Technology, Jiangxi Agricultural University, Nanchang, China; 2 Jiangxi Provincial Key Laboratory for Animal Health, College of Animal Science and Technology, Jiangxi Agricultural University, Nanchang, China

**Keywords:** neonatal piglet diarrhea, rotavirus, isolation and identification, monoclonal antibodies, indirect ELISA method

## 1 Introduction

Porcine rotavirus (PoRV) was a major pathogen causing severe diarrhea and high mortality in young piglets globally, leading to significant economic losses (1). The disease had a short incubation period (16–24 h) and was widespread in China with a high incidence and mortality (2). Infected piglets often exhibited vomiting, diarrhea, dehydration, acid-base imbalance, and death from secondary infections (3), frequently involving mixed viral serotypes (4). Older pigs typically show subclinical infections (5). Developing accurate PoRV diagnostics was considered crucial.

Clinical symptoms and epidemiology (seasonality, high piglet susceptibility) overlapped significantly with other enteric coronaviruses, including Transmissible Gastroenteritis Virus (TGEV), Porcine Epidemic Diarrhea Virus (PEDV), and Porcine Deltacoronavirus (PDCoV), which complicated diagnosis (6). Misdiagnosis delayed treatment, potentially causing mortality exceeding 80%. Co-infections or secondary pathogens occurred in 34%−67% of cases, further hindering diagnosis based solely on symptoms or pathology (7). The current reliance on PCR and virus isolation faced challenges such as technical complexity, long turnaround times, and false-negative risks (8), which impeded real-time monitoring and control.

PoRV was a non-enveloped, double-stranded RNA virus (Reoviridae family, Rotavirus genus) with 11 genes encoding 6 structural and 6 non-structural proteins (9). Its wheel-like structure (60–80 nm diameter) consisted of an outer capsid made of VP7 and VP4; VP6 determined the serotype. Of the 10 rotavirus groups (A–J), groups A, B, C, E, and H were known to infect pigs. Group A PoRV (RVA) was the most prevalent globally (61%−74% of cases) and a primary cause of severe diarrhea in piglets and infants, posing cross-species transmission risks (10–12). In pigs, RVA mainly affected suckling to weaned piglets (13, 14).

PoRV genotyping, based on VP7 (G-type) and VP4 (P-type), showed diverse genomic combinations (15, 16). G9 was a predominant global strain, reported in China and elsewhere (17). While G5 PoRV had been detected in Jiangxi Province, China, no other serotypes had been isolated there prior to this study.

To address diagnostic challenges, this study analyzed anal swabs from diarrheic piglets on a farm in Fuzhou, Jiangxi. PCR-confirmed PoRV-positive samples were used for virus isolation in MA104 cells. This process successfully isolated Group A G5 and G9 RVA strains. The VP4 and VP7 genes were amplified and sequenced.

Subsequently, high-purity recombinant VP6 protein was produced using a prokaryotic expression system. Monoclonal antibodies (mAbs) against VP6 were generated via hybridoma technology. Leveraging the high conservation of VP6 across Group A, B, and C PoRV (18), an indirect ELISA-based rapid detection method was established. This method detected multiple PoRV serotypes simultaneously, overcoming the limitations of traditional genotyping that required multiple PCRs or sequencing (19). Moreover, the ELISA method itself offered advantages such as shorter detection time, simple procedure, and a low false-positive rate.

This research enriched PoRV genotypic data for Jiangxi Province and provided a vital scientific foundation for improved PoRVD prevention, diagnosis, and the future development of multigenotypic vaccines.

## 2 Materials and methods

### 2.1 Diarrheic samples and related viruses

Four anal swab samples (designated R1, R2, R3, R4) from diarrheic piglets were collected from a large-scale pig farm in Fuzhou City, Jiangxi Province, China. Positive controls for porcine diarrhea-associated viruses—porcine rotavirus (PoRV), porcine epidemic diarrhea virus (PEDV), transmissible gastroenteritis virus (TGEV), and porcine deltacoronavirus (PDCoV)—were stored at −80 °C in the Preventive Veterinary Laboratory of Jiangxi Agricultural University. Negative controls consisted of PCR reactions without complementary DNA (cDNA) templates (cDNA components replaced with ddH2O).

### 2.2 Main cells, strains, and antibodies

Rhesus monkey fetal kidney cells (MA104 cells), mouse myeloma cells (Sp2/0 cells), the prokaryotic expression vector pET-30a, DH5α, *E. coli BL21(DE3)*Plyss host strains, rabbit polyclonal antibodies (PAb) against rotavirus VP6 protein, Porcine rotavirus type G5 (PQ34381.1) and G11 (PQ800265.1) strains were preserved by the Preventive Veterinary Laboratory of Jiangxi Agricultural University.

### 2.3 Viral detection and isolation

Fecal swabs from diarrheic piglets were homogenized in PBS, processed by triple freeze-thaw cycles and centrifugation. RNA was extracted for cDNA synthesis and multiplex PCR screening. PoRV-positive filtrates were trypsin-activated and inoculated onto MA104 monolayers. Cells were maintained in serum-free DMEM with trypsin. Viruses were harvested after CPE appeared, passaged until stable CPE (P8), and confirmed by IFA/RT-PCR. The full length of viral genome of VP4 and VP7 were amplified by RT-PCR using the following primers (20), PoRV-VP4F:ATGGCTTCTCTAATTTACAG, PoRV-VP4R:TTATAATCTACATTGTAGTATAAGTTGTT; PoRV-VP7F:CGACTGGCTATCGGATAGCTCCTT and PoRV-VP7R:GGTCACATCATACAATTCTAAC. The VP4 and VP7 gene PCR products were inserted into pMD18-T vectors for sequencing.

### 2.4 Genetic characterization

Sequencing and BLAST analysis identified homology with GenBank strains. Using Mega 11.0 software, a phylogenetic tree of the VP4 and VP7 amino acid sequences from the rotavirus isolates is constructed using the Maximum Likelihood method (with three independent bootstrap replicates) to determine the serotypes.

### 2.5 Construction and purification of the prokaryotic expression vector for PoRV VP6 protein

The PoRV VP6 gene was cloned into pET-30a and transformed into *E. coli BL21(DE3)* Plyss. Transformed cells were grown in kanamycin-LB to OD600 0.6–0.8, then induced with 1 mmol/L IPTG for 5 h. Cells were harvested, lysed ultrasonically, and fractionated by centrifugation. His-tagged VP6 was purified from soluble fractions using nickel affinity chromatography, with expression and specificity confirmed by SDS-PAGE and anti-His Western blot.

### 2.6 Animal immunization, antibody detection and hybridoma generation

Three 8-week-old female BALB/c mice were immunized subcutaneously with VP6 emulsified in Freund's adjuvants (200 μg/dose). After assessing high serum antibody titers via ELISA, mice received a final adjuvant-free intraperitoneal booster. Splenocytes were fused with SP2/0 cells (5:1) using PEG. Hybridomas were selected in HAT medium, screened by ELISA, and subcloned to monoclonality via limiting dilution (21). Stable lines were expanded and cryopreserved.

### 2.7 mAbs validation by IFA

MA104 cells infected with trypsin-activated PoRV strains G5 (isolated in this study), G9, and G11 (both maintained by Jiangxi Agricultural University's Laboratory of Preventive Veterinary Medicine), showed 80% CPE, were fixed (4% paraformaldehyde), permeabilized (1% Triton X-100), and blocked (5% skim milk). Cells were incubated with mAbs, followed by FITC-labeled secondary antibody and DAPI. Fluorescence microscopy confirmed PoRV antigen localization (FITC-green) and nuclear staining (DAPI-blue). mAbs isotypes (IgG1, IgG2a, IgG2b, IgG3, IgA, IgM) and light chains (κ/λ) were determined using Suzhou Boao Long Technology Co., Ltd. commercial ELISA kit (BF16001). The criteria are defined as follows: Positives require OD_450_ ≥ 0.8; negatives ≤ 0.15. Positive OD_450_ must exceed negative by ≥0.15. Negative OD_450_ < 0.05 is considered 0.05.

### 2.8 Indirect ELISA development

The optimal reaction conditions for the indirect ELISA were developed using a matrix method, optimizing multiple parameters according to the standard protocol (22): coating concentration of purified VP6 protein (1.0, 2.0, 4.0, 8.0, 16.0 μg/ml), dilution factors for monoclonal antibody and negative mouse serum (1:5,000, 1:10,000, 1:20,000, 1:40,000), blocking solution (1% BSA, 2% BSA, 5% skim milk, 5% fetal bovine serum), blocking time (30, 45, 60, 75 min), dilution of HRP-conjugated goat anti-mouse IgG (1:1,000, 1:5,000, 1:7,500, 1:10,000), and incubation time (30, 45, 60, 75). Using these optimized conditions, the method's sensitivity was evaluated by testing 10-fold serial dilutions of PoRV-positive porcine serum (from 1:100 to 1:6,400), with each dilution tested in duplicate, to determine the highest positive dilution. Furthermore, the established ELISA method was used to detect antibodies in 190 clinical porcine serum samples, and the results were compared with those obtained using Shenzhen Zhenrui Biotech's Porcine Rotavirus Antibody Detection Kit (100714) to calculate the concordance rate between the two methods.

## 3 Descriptive results

**Figure 1:**
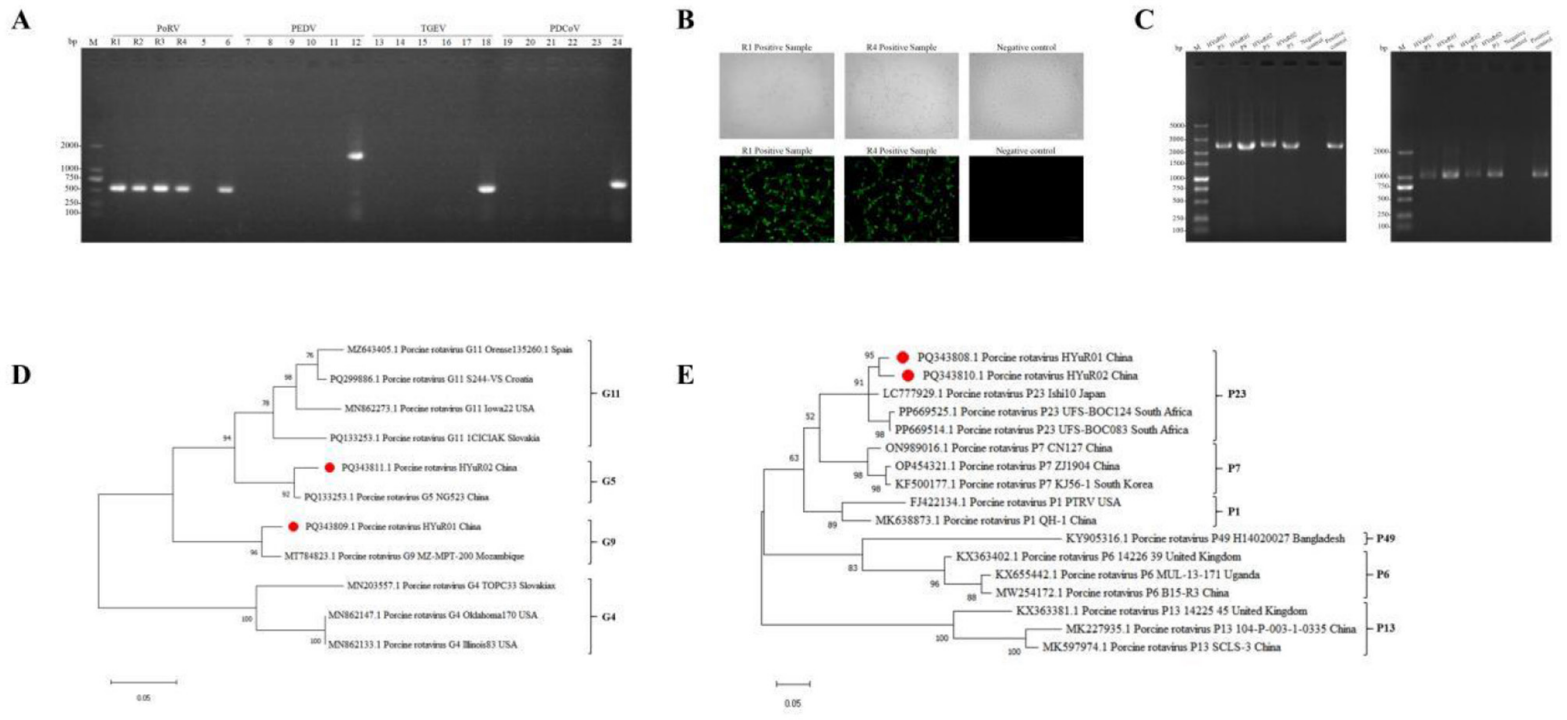
Virus isolation, phylogenetic tree construction and sequence analysis. This figure provides an overview of the virus isolation, phylogenetic tree construction, and sequence analysis, with all experiments performed in three independent replicates to ensure statistical robustness. **(A)** RT-PCR detection results of diarrhea-associated viruses in fecal samples. M: DL2000 DNA Marker; R1~R4: Diarrheal Samples; 5/11/17/23: Negat0ive control; 6/12/18/24: Positive control of PoRV, PEDV, TGEV and PDCoV. **(B)** CPE and IFA Analysis of MA104 Cells Inoculated with Filtrate from Sample R1 and R4 at the Third Passage(40×). **(C)** RT-PCR successfully amplified the VP4 and VP7 genes from both the P3 and P8 passages of HYuR01 and HYuR02 isolates, demonstrating that these viral strains are well-adapted to cell culture and capable of stable propagation through serial passages. **(D, E)** Phylogenetic analyses based on the VP7 and VP4 genes were performed to determine the G and P genotypes of the HYuR01 and HYuR02 isolates, respectively. The experimental strains are marked in red.

RT-PCR analysis of diarrheal piglet feces amplified a specific 503-bp band for PoRV. While positive controls for PEDV, TGEV, and PDCoV showed their expected bands, both the fecal sample and the ddH_2_O negative control tested negative for these viruses (Figure 1A), where M is marker, R1–R4 are diarrheal samples, lanes 5/11/17/23 are negative controls, and lanes 6/12/18/24 are positive controls for PoRV, PEDV, TGEV, and PDCoV respectively. This confirms the sample was positive for PoRV. Sequencing of PoRV-positive diarrheal samples revealed identical nucleotide sequences for R1–R3 (indicating a single strain), while R4 exhibited a distinct genetic profile. Following trypsin treatment and inoculation of R1 and R4 filtrates into MA104 cells, significant CPE emerged at the third blind passage, characterized by cell rounding, cytoplasmic granularity, and large vacuole formation. IFA of passage 3 virus demonstrated specific green fluorescence within the cytoplasm of inoculated MA104 cells (Figure 1B), whereas uninfected controls exhibited no fluorescence and maintained normal morphology. RT-PCR analysis of P3 and P8 viral passages demonstrated amplification of specific bands corresponding to the VP7 (1,035 bp) and VP4 (2,331 bp) genes, confirming stable viral propagation across serial passages (Figure 1C). Based on these findings, isolates R1 and R4 were designated as HYuR01 and HYuR02, respectively. VP7/VP4 genes of isolates HYuR01/HYuR02 were sequenced and uploading to NCBI (NCBI: PQ343808.1-PQ343811.1). Phylogenetics showed HYuR01 VP7 had 96% aa identity with Chinese G9 (MT784823.1), confirming G9 genotype. HYuR02 VP7 shared 92% identity with Chinese G5 (PQ133253.1), designating it G5 (Figure 1D). Both shared 91% VP4 identity with Japanese P23 (LC777929.1), grouping as P23 (Figure 1E). High divergence suggests intercontinental reassortment, necessitating updated diagnostics and multigenotypic vaccines.

**Figure 2:**
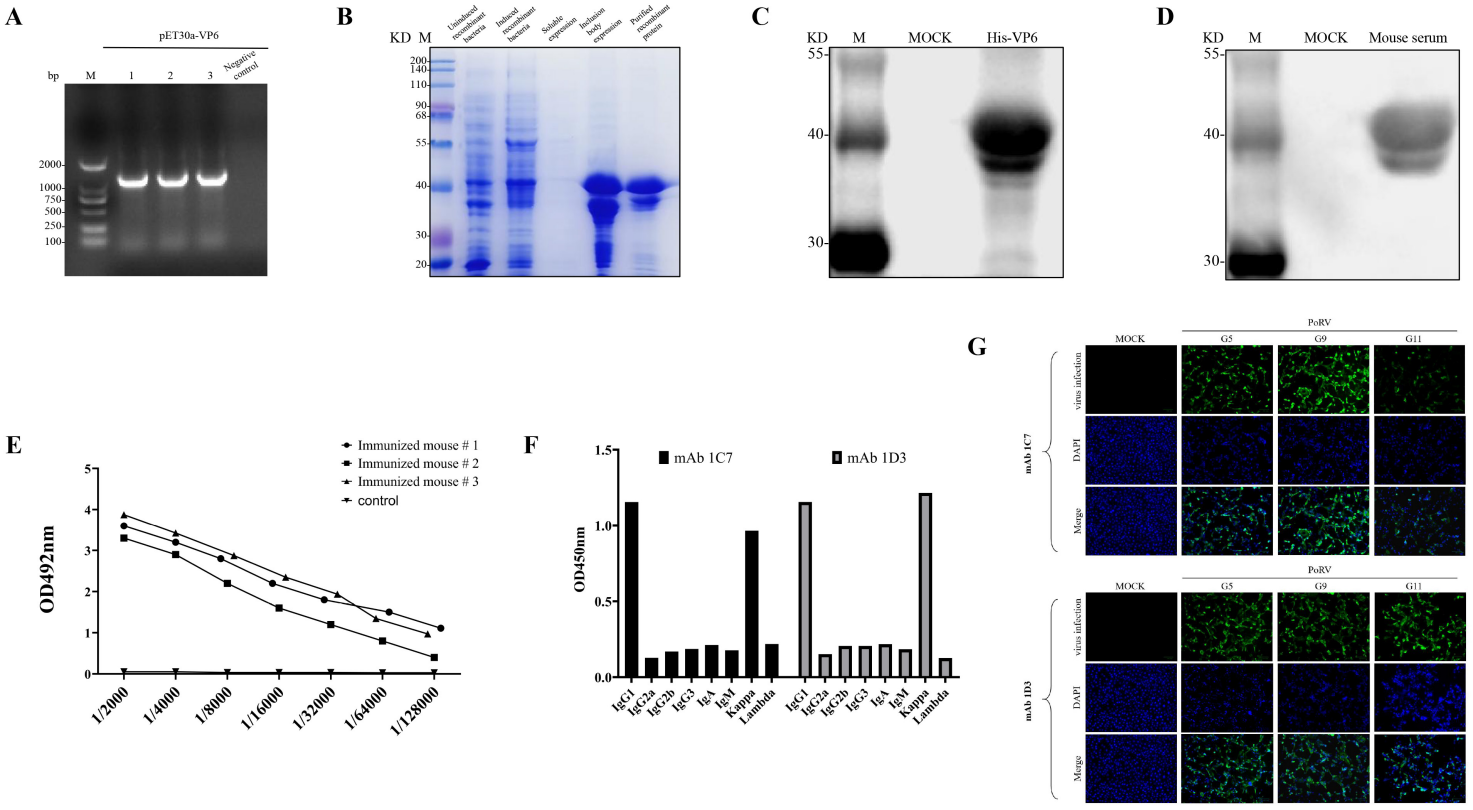
Preparation of Monoclonal Antibodies Against Rotavirus VP6 Protein. This figure illustrates the construction of the pET30a-VP6 prokaryotic expression vector, protein purification, animal immunization, serum antibody detection, and the application of mAbs 1C7/1D3 in indirect immunofluorescence assays, with all experiments conducted in three independent replicates to ensure statistical robustness. **(A)** Colony PCR Verification of the pET-30a-VP6 Recombinant Plasmid. **(B)** Induced Expression and Protein Purification of the Recombinant Strain pET-30a-VP6-BL21(DE3)Plyss. **(C)** Recombinant VP6 protein was identified using an anti-His monoclonal antibody. **(D)** Recombinant VP6 protein was verified with PoRV-positive serum. **(E)** Serum antibody titers were determined via indirect enzyme-linked immunosorbent antibody assay(ELISA). **(F)** Isotype and Subclass Characterization of mAbs 1C7/1D3. **(G)** Fluorescent Reactions of mAbs 1C7/1D3 to Three Genotypes of PoRV (100 μm).

Specific amplification of the VP6 gene (1206 bp) of the G9 genotype HYuR01 strain was performed, and the gene was cloned into the pET30a vector (Figure 2A). colony PCR confirmed positive bands corresponding to the target insert. Subsequent Sanger sequencing verified that the inserted sequence in the recombinant plasmid exhibited complete identity with the target VP6 gene, with no frameshift or missense mutations. A new recombinant plasmid was obtained, named pET30-VP6. Coomassie Brilliant Blue R-250 staining revealed that the recombinant strain pET30a-VP6-BL21(DE3)Plyss expressed a protein of approximately 40 kDa post-induction, consistent with the predicted molecular weight of VP6. The recombinant protein was predominantly expressed in inclusion bodies. Following purification via His-tag nickel affinity chromatography, SDS-PAGE analysis demonstrated a single band corresponding to VP6, indicating high purity (Figure 2B). Western blot confirmed specific recognition of the recombinant VP6 protein by both anti-His monoclonal antibodies (Figure 2C) and post-immunization mouse serum (Figure 2D), confirming successful expression and robust immunogenicity of the recombinant protein. Following the third immunization, serum was collected via tail vein bleeding and anti-VP6-specific antibody titers were measured by indirect ELISA. All immunized mice exhibited antibody titers ≥1:64,000 (Figure 2E). The mouse with the highest titer (1:128,000) was selected for splenocyte isolation and subsequent PEG-mediated fusion with SP2/0 myeloma cells to generate hybridoma cell lines. Positive hybridoma clones secreting anti-PoRV VP6 antibodies were selected via indirect ELISA screening. Three rounds of limiting dilution subcloning were performed to ensure monoclonality, yielding two stable hybridoma cell lines designated as mAbs 1C7/1D3. Immunoglobulin subclass analysis revealed that both mAbs possess kappa light chains and belong to the immunoglobulin (Ig) G1 isotype (Figure 2F). The MA104 cells infected with PoRV were subjected to indirect immunofluorescence assay (IFA) following standardized protocols. Both monoclonal antibodies 1C7/1D3 exhibited intense and specific fluorescence signals localized within the cytoplasm of infected cells, confirming their capability to detect PoRV antigens across three G-genotypes (Figure 2G). No fluorescent signals were observed in uninfected control groups, demonstrating the specificity of this detection method. These results substantiate the value of these monoclonal antibodies as reliable diagnostic tools for detecting multiple genotypes of porcine rotavirus through IFA.

Matrix titration experiments established optimal conditions for an indirect ELISA using mAb 1C7. PoRV VP6 antigen was coated at 4 μg/mL for 12–16 h at 4 °C. Blocking used 5% skimmed milk for 60 min at 37 °C. The mAb 1C7 primary antibody was diluted 1:20,000 and incubated for 60 min at 37 °C. The goat anti-mouse IgG HRP conjugate secondary antibody was applied at 1:5,000 dilution for 45 min at 37 °C. This established method demonstrated good repeatability. Testing eight PoRV-positive porcine sera in duplicate on plates from the same batch yielded an intra-assay coefficient of variation CV below 10 percent. Testing the same sera on plates from different batches yielded an inter-assay CV also below 10 percent. Furthermore, the method's clinical reliability was confirmed. When testing 190 porcine serum samples alongside the Shenzhen Zhenrui Biotech Porcine Rotavirus Antibody Detection Kit lot 100714, the developed ELISA detected 169 positives and 21 negatives, an 88.9 percent positive rate. The commercial kit detected 175 positives and 15 negatives, a 92.1 percent positive rate. The 96.5 percent concordance rate between methods confirmed the established ELISA's suitability for clinical detection (Table 1).

**Table 1:** Clinical sample test results (Total Samples Tested: 190 porcine serum).

**Parameter**	**The developed ELISA method**	**Commercially available kit**	**Concordance rate**
Positives	169	175	96.5%
Negatives	21	15	
Detection rate	88.9%	92.1%	

Overall, this study reports the isolation of a G9-type PoRV strain in Jiangxi Province, alongside the development of a high-sensitivity recombinant VP6 protein and broadly reactive monoclonal antibodies. Furthermore, a reliable ELISA detection method is established as an effective alternative to commercial kits. These advancements provide critical technical support for enhanced epidemiological surveillance and targeted control of PoRV.

## Data Availability

The original contributions presented in the study are included in the article/supplementary material, further inquiries can be directed to the corresponding author.
