# Correction: Isolation, identification of porcine a rotavirus, and preparation of the monoclonal antibodies

**DOI:** 10.3389/fvets.2025.1749139

**Published:** 2025-12-18

**Authors:** Yu Hu, Hongjin Wu, Boqian Zha, Deping Song, Xiangdong Wu, Huansheng Wu

**Affiliations:** 1 Department of Veterinary Preventive Medicine, College of Animal Science and Technology, Jiangxi Agricultural University, Nanchang, China; 2 Jiangxi Provincial Key Laboratory for Animal Health, College of Animal Science and Technology, Jiangxi Agricultural University, Nanchang, China

**Keywords:** neonatal piglet diarrhea, rotavirus, isolation and identification, monoclonal antibodies, indirect ELISA method

There was a mistake in Figure 2G as published.

The IFA image intended to show “the identification of fluorescent response in MA104 cells infected with G5 porcine rotavirus using mAb 1D3” was unintentionally misplaced from another experiment. This was solely due to a mislabeling of image files during figure assembly and does not affect the experimental results, underlying data, or the scientific conclusions of our study in any way. It was an inadvertent presentational oversight. The corrected figure appears below.

**Figure 2:**
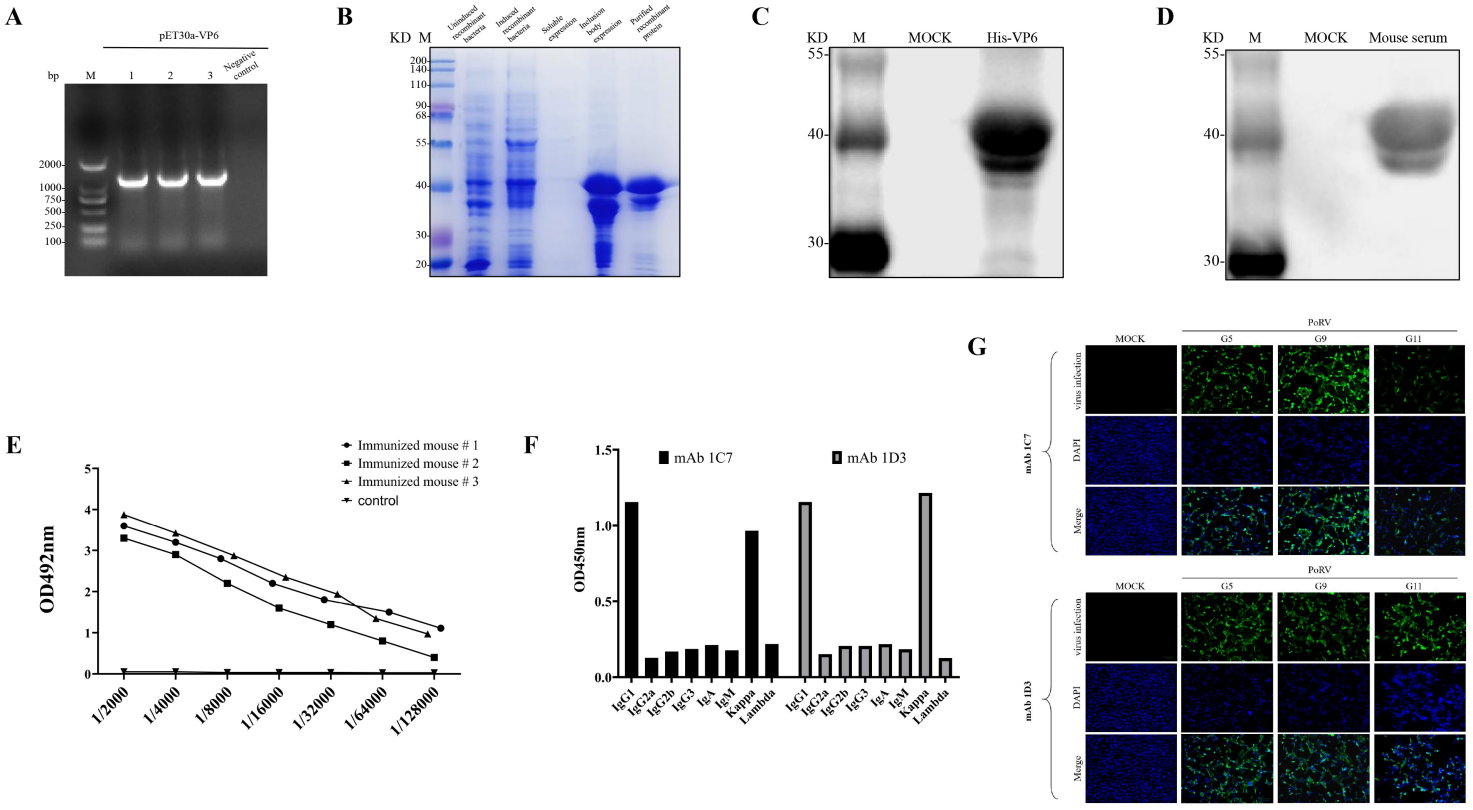
Preparation of monoclonal antibodies against rotavirus VP6 protein. This figure illustrates the construction of the pET30a-VP6 prokaryotic expression vector, protein purification, animal immunization, serum antibody detection, and the application of mAbs 1C7/1D3 in indirect immunofluorescence assays, with all experiments conducted in three independent replicates to ensure statistical robustness. **(A)** Colony PCR Verification of the pET-30a-VP6 Recombinant Plasmid. **(B)** Induced Expression and Protein Purification of the Recombinant Strain pET-30a-VP6-BL21(DE3)Plyss. **(C)** Recombinant VP6 protein was identified using an anti-His monoclonal antibody. **(D)** Recombinant VP6 protein was verified with PoRV-positive serum. **(E)** Serum antibody titers were determined via indirect enzyme-linked immunosorbent antibody assay (ELISA). **(F)** Isotype and Subclass Characterization of mAbs 1C7/1D3. **(G)** Fluorescent Reactions of mAbs 1C7/1D3 to Three Genotypes of PoRV (100 μm).

Alt-text for Figure 2 has been modified to align with this correction.

The original version of this article has been updated.

